# Cyclic Crack Growth in Chemically Tailored Isotropic Austenitic Steel Processed by Electron Beam Powder Bed Fusion

**DOI:** 10.3390/ma14216544

**Published:** 2021-11-01

**Authors:** Matthias Droste, Ruben Wagner, Johannes Günther, Christina Burkhardt, Sebastian Henkel, Thomas Niendorf, Horst Biermann

**Affiliations:** 1 Institute of Materials Engineering, Technische Universität Bergakademie Freiberg, Gustav-Zeuner-Straße 5, 09599 Freiberg, Germany; matthiasdroste@web.de (M.D.); christina.burkhardt@iwt.tu-freiberg.de (C.B.); henkel@ww.tu-freiberg.de (S.H.); biermann@ww.tu-freiberg.de (H.B.); 2 Institute of Materials Engineering, Universität Kassel, Mönchebergstraße 3, 34125 Kassel, Germany; johannes.guenther1988@web.de (J.G.); niendorf@uni-kassel.de (T.N.)

**Keywords:** additive manufacturing, threshold value, TRIP, isotropic microstructure

## Abstract

The present study analyzes the cyclic crack propagation behavior in an austenitic steel processed by electron beam powder bed fusion (PBF-EB). The threshold value of crack growth as well as the crack growth behavior in the Paris regime were studied. In contrast to other austenitic steels, the building direction during PBF-EB did not affect the crack propagation rate, i.e., the crack growth rates perpendicular and parallel to the building direction were similar due to the isotropic microstructure characterized by equiaxed grains. Furthermore, the influence of significantly different building parameters was studied and, thereby, different energy inputs causing locally varying manganese content. Crack growth behavior was not affected by these changes. Even a compositional gradation within the same specimen, i.e., crack growth through an interface of areas with high and areas with low manganese content, did not lead to a significant change of the crack growth rate. Thus, the steel studied is characterized by a quite robust cyclic crack growth behavior independent from building direction and hardly affected by typical parameter deviations in the PBF-EB process.

## 1. Introduction

Austenitic stainless steels are widely used in industry due to their outstanding corrosion resistance and their combination of high ultimate tensile strength and excellent ductility. However, after being processed by additive manufacturing (AM), e.g., by using powder bed fusion techniques like selective laser melting (PBF-LB, also SLM) or electron beam melting (PBF-EB, also EBM), these steels are often characterized by columnar grains and a strongly anisotropic crystallographic texture [1,2,3,4,5,6,7]. Demanding and adapted process strategies, finally aiming at adjusting the solidification rate as well as the direction and size of the temperature gradient during processing, are usually needed to obtain isotropic microstructures [3,8,9]. In 2018, Günther et al. [10] published a study focusing on a CrMnNi steel with a nominal composition of X5CrMnNi16-7-6 processed by PBF-EB overcoming this issue. The deformation mechanisms and properties of this steel processed by conventional techniques were already studied in depth, see, e.g., [11,12,13,14,15,16,17,18]. In contrast to other austenitic steels like AISI 316L, the CrMnNi steel exhibits a primary ferritic solidification leading to a solid–solid phase transformation bcc→fcc upon cooling [10]. Due to adjacent scan tracks and further layers added on top, i.e., the layer-wise PBF-EB production principle, the material gets heated up several times [10]. This process’ inherent cyclic heat treatment provokes repetitive phase transformations and is supposed to induce an isotropic microstructure with nearly equiaxed grains of about 30 µm size and without a pronounced crystallographic texture [10]. No special scan strategy is needed, i.e., this alloy offers a more robust and more reliable approach to achieve materials with isotropic properties after additive manufacturing.

Different aspects of this PBF-EB processed CrMnNi steel have been investigated and published so far. Besides the influence of different processing strategies [10,19] on microstructure evolution, studies have focused on the quasi-static properties [10,19]; the cyclic deformation behavior, which has been investigated in total strain controlled fatigue tests [20]; feasibility analyses considering different kinds of square-celled structures and their mechanical behavior during compression testing [21,22]; and the influence of a process-induced variation in manganese fraction on the tensile properties [19]. The latter was achieved by adjusting the beam parameters and energy input, respectively, and, thereby, varying the extent of evaporation of manganese during PBF-EB processing. A higher energy input led to more pronounced Mn evaporation and, thus, a lower manganese content after PBF-EB [19]. All studies have been accompanied by in-depth analysis of the microstructure and assessment of the most relevant deformation mechanisms.

An aspect still missing up to now is characterization of the cyclic crack growth behavior. It is known from other AM austenitic steels that the crack propagation rate parallel to the building direction differs from the crack propagation rate perpendicular to the building direction [7,23,24,25,26] due to the anisotropic microstructure and, in particular, due to the columnar grains aligned along the building direction. Usually, the crack growth rates are higher parallel to the columnar grains, i.e., parallel to the building direction. As the present steel exhibits an isotropic microstructure, the crack growth behavior in different directions is an interesting matter, which will be addressed in the present work. Further points of focus are the effect of significantly different beam parameters and energy inputs on the crack growth behavior as well as the effect of a chemical gradation, i.e., crack propagation, through interfaces of areas with high and areas with low manganese content.

## 2. Materials and Methods

**Figure 1:**
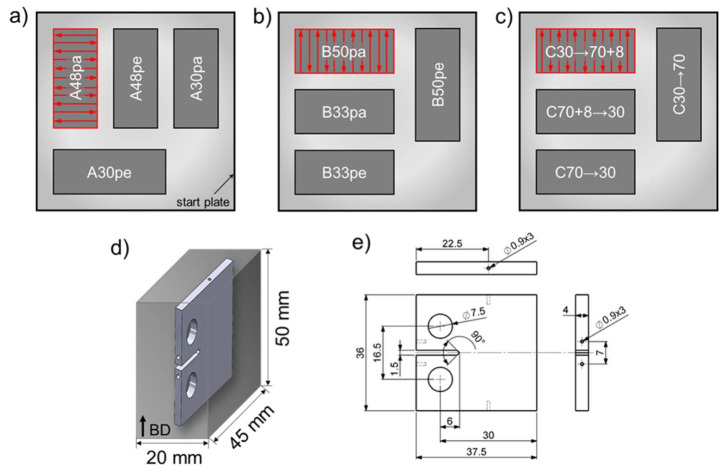
Arrangement of the blocks during PBF-EB processing of batches A (**a**), B (**b**), and C (**c**) as well as a schematic detailing extraction of a miniature CT-specimen from a block ((**d**), CGD ⊥ BD) and geometry of miniature CT-specimens ((**e**), dimensions in mm).

The steel powders for PBF-EB and hot pressing (HP) as a reference technology were gas atomized by TLS (Bitterfeld, Germany). Chemical compositions and characteristic values for the particle size distribution are given in Table 1. An Arcam A2X (Arcam EBM, Mölnlycke, Sweden) PBF-EB machine was operated under a controlled atmosphere, i.e., a helium pressure of 2 × 10^−3^ mbar. A 100 mm × 100 mm × 10 mm start plate made of stainless steel 1.4301 was heated up to the initial temperature of 850 °C. Rectangular blocks with a cross section of 45 mm × 20 mm and a height of 50 mm were manufactured by PBF-EB. A meander-shape scan strategy was used accompanied by a rotation of the scan direction of 180° between each layer. Thus, the length of every scan path was set constant at 20 mm, see Figure 1a. The arrangement of the blocks on the 100 mm × 100 mm start plate and their designation is shown in Figure 1a. For batches A and B, each beam parameter set was used for two blocks to enable extraction of CT-specimens with crack growth direction (CGD) perpendicular and parallel to the building direction. For batches A and B, acceleration voltage *U_A_*, beam current *I_B_*, and layer thickness *t* of 60 kV, 7.5 mA, and 50 µm, respectively, were used. In order to vary the volume-energy *E_vol_* between the individual blocks, i.e., the energy input per volume unit, different scan speeds *v_s_* and hatch distances *l* were applied. The resultant volume energies were in the range of 30 J/mm^3^ to 50 J/mm^3^. Table 2 provides an overview of the relevant parameters. The nomenclature considers the batch number followed by the volume energy and crack growth direction (parallel (pa) or perpendicular (pe) to the build direction BD, respectively).

The beam parameters for the blocks of batch C were changed during PBF-EB processing after finishing the first half, i.e., after building the first 25 mm. The two halves were manufactured using significantly different volume energies in order to investigate the influence of such a change in building conditions and the highly affected final Mn content, respectively, on the crack growth behavior. In case of the material states C30 → 70 + 8 and C70 + 8 → 30 one half of the blocks was exposed to energy input by the electron beam on each built layer twice, first with an energy of 70 J/mm^3^ and a second time with an energy of 8 J/mm^3^. This procedure was carried out to maximize the Mn gradation, see Table 2. The different nomenclatures indicate the later crack growth directions.

For the reference material, the conventional sintering technology of HP was used to produce compact discs. In a first step, the powder was pressed to a green body at 60 MPa under laboratory atmosphere and room temperature. The subsequent HP process was carried out under vacuum. In the beginning, the temperature was increased up to 1250 °C at a heating rate of 10 K/min. After reaching the maximum temperature, a uniaxial pressure of 30 MPa was applied via an actuator. Pressure and temperature were kept constant for 30 min. Finally, the pressure was slowly reduced and the temperature was lowered at a cooling rate of about 5 K/min. The resulting cylindrical discs exhibited a diameter of 150 mm and a height of 20 mm.

Miniature CT-specimens with a width *W* of 30 mm and a thickness *B* of 4 mm were manufactured by electro-discharge machining. All relevant geometric dimensions are shown in Figure 1e. Three specimens were extracted from each PBF-EB block (Figure 1d). The crack growth experiments were carried out on a Rumul Mikrotron resonance testing machine (Russenberger Prüfmaschinen AG, Neuhausen am Rheinfall, Switzerland) with a resonance frequency between 63 Hz and 76 Hz depending on the crack length. To avoid effects of the notch on the crack growth rate, a starting crack with a length of 1 mm was introduced prior to the actual test by cycling at a constant cyclic stress intensity factor Δ*K* of 14 MPa$\sqrt{m}$ at a load ratio of R = 0.1. Due to the limited dimensions of specimens provided by PBF-EB, R = 0.1 was chosen to ensure no plasticization of the ligament. Δ*K* decreasing tests were started at 14 MPa$\sqrt{m}$ and the load was decreased continuously with increasing crack length with the following relationship:

(1) $$∆K_{I}=∆K_{I,0}\cdot e^{C\cdot \left(a-a_{0}\right)}$$

where *C* is the reduction rate and Δ*K_I_*_,0_ and *a*_0_ are the cyclic stress intensity factor and crack length at the beginning of load reduction, respectively. In all Δ*K* decreasing tests the reduction rate was set to *C* = −0.2.

In case of the CT-specimens manufactured by a single parameter set (batches A and B) or by HP, the crack length was measured by the direct current potential drop method using a Matelect DCM-2 (Matelect Ltd., Harefield, UK) crack growth monitor. For this purpose, solder pins with a diameter of 1 mm were inserted in the drilled holes of the specimens, see Figure 1e. A pulsed direct current of 2.5 A was introduced via the vertical pins, whereas the potential above and below the notch was measured at the horizontal pins above and below the notch. The resulting voltage *U*_1_ was compared to a voltage *U*_0_ measured on a non-cracked reference specimen prepared in the same way and connected in series to the test specimen. The quotient *U*_1_*/U*_0_ was used for calculation of the crack length *a* based on a previously determined calibration curve that specifies the correlation between *a/W* and *U*_1_*/U*_0_. Electric insulation of the CT-specimens was achieved by using zirconia pins with a diameter of 7.2 mm for installation in the clevis used for mounting. After the tests, the length of the notch was measured by light optical microscopy. The same was done for the crack length at reaching the threshold value Δ*K_th_*. In this case, the specimens were finally broken by applying a quasi-static overload allowing for the assessment of the marker line related to the crack length at Δ*K_th_* on the fracture surface. The crack length measurement was corrected according to these values.

The use of the direct current potential drop method was not applicable in the case of the graded specimens (batch C) as the gradation seemed to influence the signal and, thereby, impaired the calculated crack length. Therefore, the indirect potential drop method using crack gauges of type Krak Gage A10 (Russenberger Prüfmaschinen AG, Neuhausen, Switzerland) was applied for crack length measurement instead. At least one crack gage was fixed to the surface of the CT-specimens. Due to the limited gage length of the crack gages of 10 mm, in some cases a second crack gage was applied with a slight offset on the other side of the CT-specimens. The crack reached the second crack gage after a few millimeters of propagation. This way, the monitoring and recording of the crack length could be continued after exceeding the maximum gage length of the first crack gage. The crack gages were connected to a Fractomat (Russenberger Prüfmaschinen AG, Neuhausen, Switzerland) for analyzing the signals.

Scanning electron microscopy (SEM) investigations were carried out at a field-emission gun Tescan Mira 3 (Tescan, Brno, Czech Republic) including energy dispersive spectroscopy (EDS). The specimens were conventionally ground and polished and finally vibration polished (SiO_2_ suspension of 0.02 µm grit size) for several hours. Electron backscatter diffraction (EBSD) measurements were used for calculation of an area-weighted average grain size $\bar{d_{A}}$ according to the following relationship:

(2) $$\bar{d_{A}}=\frac{\sum_{i=1}^{N}A_{i}\cdot d_{i}}{\sum_{i=1}^{N}A_{i}}$$

where *Ai* and *di* are the area and diameter of the *i*th grain, respectively. Misorientations ≥ 15° were considered as high angle grain boundaries.

## 3. Results and Discussion

### 3.1. Microstructures after Electron Beam Powder Bed Fusion

**Figure 2:**
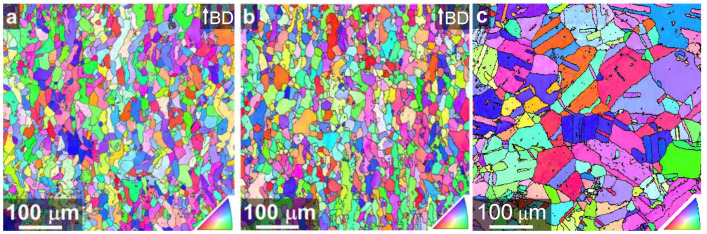
EBSD orientation maps showing the microstructure after PBF-EB processing with parameter sets B33 (**a**) and B50 (**b**). Building direction is vertical. (**c**) EBSD orientation map of HP reference material modified after [16]. High angle grain boundaries are marked black.

Figure 2 exemplarily compares the microstructures after the PBF-EB process for two parameter sets with considerably different energy inputs of 33 J/mm^3^ (Figure 2a, B33) and 50 J/mm^3^ (Figure 2b, B50). In this case, the volume energy variation was achieved solely by changing the scan speed *v_s_* from 3000 mm/s to 2000 mm/s, see Table 2. However, as already reported by Günther et al. [10] for this steel alloy, the formation of the microstructure was hardly affected by changes in the energy input as can be deduced from the EBSD orientation maps (Figure 2). The area-weighted average grain sizes were 24 µm and 26 µm and the grain shape aspect ratios were 0.40 and 0.39 for the parameter sets B33 and B50, respectively. Thus, both parameter sets resulted in a fine-grained microstructure of very similar appearance characterized by slightly elongated grains along the building direction. Based on these results and data from the literature [10,19,21], it is assumed that all other material states processed by PBF-EB in the present work exhibited similar, narrow grain size distribution and similar grain morphology.

The HP reference material, on the other hand, exhibited a significantly higher area-weighted average grain size of 62 µm (Figure 2c). For further analyses of the reference material the reader is referred to [16].

### 3.2. Influence of Building Direction

**Figure 3:**
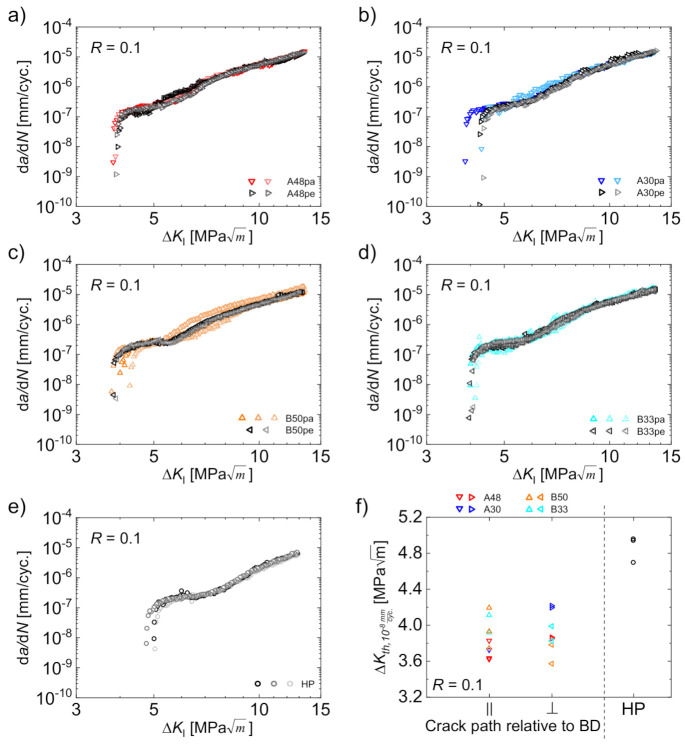
Crack growth rates for crack growth direction parallel and perpendicular to the building direction for parameter sets A48 (**a**), A30 (**b**), B50 (**c**), and B33 (**d**) as well as crack growth rates for the HP reference material (**e**); corresponding threshold values Δ*K_th_*_,10_^−8^_mm/cyc._ for all conditions (**f**).

Firstly, the influence of the building direction of the PBF-EB process on the crack growth rate d*a*/d*N* was assessed. For this purpose, the crack growth curves of the Δ*K* decreasing tests were plotted in Figure 3a–d comparing parallel and perpendicular crack growth directions for each parameter set. The equivalent curves for the HP reference material are shown in Figure 3e. At least two CT-specimens were tested for each condition to assess repeatability. All three curves for the reference material revealed almost identical crack growth rates with only minor differences in the threshold region (Figure 3e). Even though the scatter for each individual PBF-EB parameter set was slightly higher (Figure 3a–d), it was still considered to be low, i.e., the velocity of crack growth seems to be independent from the building direction during PBF-EB. Rather, the small deviations were attributed to well-known sample to sample variations and scatter, respectively, than to a noteworthy difference originating from the crack growth direction. This statement can be affirmed by discussing the material states B50pa and B50pe in Figure 3c in more detail. In this case, the most pronounced scatter was seen for two specimens exhibiting a crack path parallel to the building direction (B50pa). In contrast, the curves for the crack growth perpendicular to the building direction (B50pe) were located in between the former. Thus, the differences do not reflect any effect related to different building directions but rather arbitrary scatter. The same holds true for the threshold values, those values evaluated at a crack growth rate of 10^−8^ mm/cycle Δ*K_th_*_,10_^−8^_mm/cyc__._, which are depicted in Figure 3f for all material states. All data points for the PBF-EB processed specimens were in a range from 3.6 MPa$\sqrt{m}$ to 4.2 MPa$\sqrt{m}$. Prefactors for deriving quantile growth curves for aluminum alloys (being also fcc) were 1.39 (5%) and 0.73 (95%), respectively, for the threshold value. Thus, the observed variations were considered as usual scatter [27]. In particular, no distinct difference between the values determined for crack growth parallel to the building direction and perpendicular to the building direction was observed. Obviously, the threshold value for the HP reference material was markedly higher (Figure 3f). For a discussion on reasons for this fact, the reader is referred to Section 3.3.

The findings highlighting crack growth characteristics independent from the building direction are in disagreement to the results of other authors [7,23,24,25,26]. The articles of Riemer et al. [7] as well as Suryawanshi et al. [23] dealt with an AISI 316L processed by PBF-LB and reported a dependence of crack growth rates and, in particular, the threshold value from the building direction. According to Riemer et al. [7], the elongated shape of the grains aligned parallel to the building direction is responsible for this behavior. Alongside the elongated grains, the crack growth is accelerated whereas it is decelerated when cracks are growing perpendicular to the longitudinal axis of the grains. In contrast to the microstructure of AISI 316L, the austenitic steel considered in the present work only exhibits a slightly elongated grain shape as discussed in Section 3.1 and as shown previously [10,21]. Instead, an isotropic microstructure with almost equiaxed grains developed due to the cyclic heat treatment inherent to the PBF-EB-process. This intrinsic type of cyclic heat treatment provokes several phase transformations in the material [10]. Thus, the difference to the two aforementioned studies [7,23], both focusing on steels with an anisotropic grain shape, had to be expected. Instead, the similar threshold values independent from the building direction (Figure 3f) indicate an isotropic fatigue crack growth behavior for the present steel, most probably induced by the almost isotropic microstructure. This in particular holds true as the microstructural influence on the cyclic crack growth is much more pronounced in the near-threshold regime compared to the Paris region [28].

### 3.3. Influence of Building Parameters

**Figure 4:**
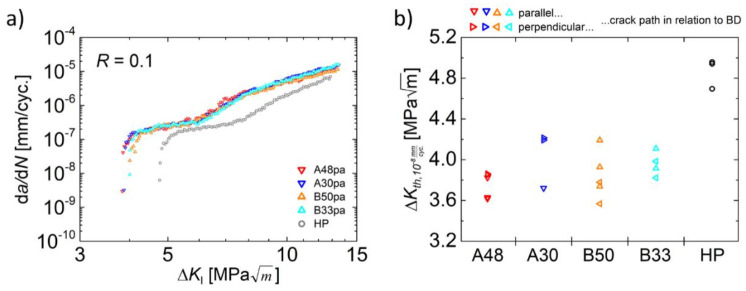
Comparison of the crack growth curves (**a**) and threshold values Δ*K_th_*_,10_^−8^_mm/cyc._ (**b**) for the individual material states.

After detailing the independence of the cyclic crack growth behavior from the building direction in the preceding section, a comparison of the individual material states is given in the following. For this purpose, one representative d*a*/d*N* curve for each parameter set of the PBF-EB process as well as for the HP reference material was plotted in Figure 4a.

Again, only minor scatter is observed when comparing the different material states processed by PBF-EB. Clearly this indicates a quite robust fatigue crack growth behavior of the steel considered here, i.e., an insensitivity towards changes of the building parameters. Even the threshold values of the different PBF-EB material states, which are shown in Figure 4b, do not reveal a systematic deviation for any of the different parameter sets. All calculated values are located in the same scatter band. Thus, even significant variations in the energy input prevail, i.e., values between 30 J/mm^3^ and 50 J/mm^3^ were applied and obviously no effect on the cyclic crack growth is present.

On the one hand, this is in accordance with Günther et al. [10], who found similar fine-grained isotropic microstructures for the present steel processed by different PBF-EB beam parameters. On the other hand, the manganese fraction after PBF-EB processing is known to differ for the present steel alloy due to evaporation [10,19]. Evaporation of elements during PBF-EB is a known phenomenon which is eventually triggered by the low pressure in the process chamber [29]. In particular, manganese is prone to evaporation (see [30]) because of its low temperature of ebullition, which at a pressure of 2 × 10^−3^ mbar (characteristic for PBF-EB) is reached already at about 860 °C [31]. Thus, it is significantly lower compared to values of other alloying elements of austenitic steels and even lower compared to aluminum, where the temperature of ebullition is about 1090 °C under these conditions [31]. However, during PBF-EB processing, the extent of evaporation can be influenced within certain limits by adjusting the beam parameters and, thus, the energy input *E_vol_* [10,19]. High beam energies lead to relatively low manganese fractions in the material processed and vice versa [10,19]. In line with these considerations, for the present material state B50, EDS measurements revealed a Mn content of 4.8 wt.%, whereas its counterpart state B33 contained 6.0 wt.% of Mn, see Table 2. Despite this difference, the cyclic crack growth behavior was not affected as shown in Figure 4a, i.e., the material behavior can be rated as quite robust and insensitive to different processing strategies as already stated with respect to the independence of crack growth from the building direction.

In contrast, the HP reference material revealed a slightly lower crack growth rate (Figure 4a) and a higher threshold value (Figure 4b) in comparison to the PBF-EB processed material states. This is attributed to the higher average grain size of the reference HP material, which is 62 µm compared to 24 µm and 26 µm in the case of the PBF-EB processed counterparts. A larger grain size is known to cause such effects at small stress intensity factor ranges [32]. In particular, a higher threshold value with increasing grain size was repeatedly reported [28,33,34,35]. A coarse-grained microstructure leads to the evolution of rougher crack surfaces and, thereby, promotes the roughness-induced crack closure effect, which, in turn, is responsible for the higher threshold value.

Another feature of all the curves shown in Figure 4a is a characteristic kink at crack growth rates d*a*/d*N* of about 2 × 10^−7^ mm/cycle and at cyclic stress intensity factors Δ*K* of about 6 MPa$\sqrt{m}$ for the PBF-EB-processed material states and of about 8 MPa$\sqrt{m}$ for the HP reference condition. A further decrease of the cyclic stress intensity factor leads to an almost plateau-like behavior, i.e., the crack growth rate d*a*/d*N* decreases only slightly with decreasing Δ*K*. After reaching Δ*K* values of 4 MPa$\sqrt{m}$ (PBF-EB conditions) and 5 MPa$\sqrt{m}$ (reference material), respectively, the curves (Figure 4a) exhibit a drop of crack growth rates, which is typically observed near the threshold value, i.e., in regime I of a common d*a*/d*N* vs. Δ*K* curve. Although the reason of the plateau-like behavior was not investigated in detail in the present work, some explanations found in the literature are considered here. Robin and Pluvinage reported, for a 2618A aluminum alloy, on the occurrence of a so-called aluminum knee similar to the plateau-like behavior in the present work. The authors rationalized the crack growth transition with a transition from an opening mode to a shear mode of fracture, while the plastic zone had no influence on this transition [36]. McEvily et al. [37,38] investigated the cyclic crack growth behavior of an AISI 304 stainless steel in air and in vacuum. They also reported on the kink and plateau-like behavior, but only for tests performed in ambient air [37]. Tests performed under vacuum, on the contrary, did not show such discontinuities [37]. In fact, the crack growth rates were slower and the threshold values were higher under vacuum conditions. Thus, some environmental effect was assumed to influence the crack propagation behavior, eventually leading to the observed plateaus in air. In general, an environmental effect on cyclic crack propagation is well-known for different metals and alloys [28,39,40,41,42,43]. It is a result of the competing effects of oxide-induced crack closure, which decreases the crack growth rate, and anodic as well as cathodic stress corrosion phenomena increasing the crack growth rate [28]. The related elementary mechanisms obviously depend on further factors such as material composition, loading frequency, or temperature [28]. In the studies of McEvily et al. [37,38], the detrimental effect stemming from atmosphere seemed to dominate. Other authors also reported a decreased threshold value for austenitic stainless steels in environments like hydrogen, moist air, or chloride solutions [44,45]. As mentioned above, the data obtained in the present work cannot finally pinpoint the most dominant mechanism responsible for the observed kink in the d*a*/d*N* curves. Nevertheless, several studies indicate an influence of corrosion processes, which are commonly known to be time dependent. The latter, in turn, explains why the environmental impact is more pronounced at decreasing crack growth rates, i.e., it is much higher in the threshold regime than in the Paris regime, see [28]. Thus, the kink is probably just a result of this time dependence. Eventually, these corrosion phenomena are thought to markedly affect the crack propagation only for a distinct crack growth level in ambient air at the given test frequency. Here, these effects lead to a decrease of the threshold value.

### 3.4. Influence of Gradation

Günther et al. [19] already investigated the effect of a chemical gradation induced by different energy inputs during PBF-EB processing on the quasi-static mechanical behavior of the present steel alloy. They reported manganese fractions between 3.3 wt.% and 6.2 wt.% based on five different PBF-EB-parameter sets considering energy densities ranging from 29.3 J/mm^3^ to 70.6 J/mm^3^ [19]. For the dependence of the manganese fraction, the authors reported on a difference in kinetics of α’-martensite formation and, eventually, strain hardening behavior and tensile strength. The latter value increased with decreasing manganese content [19]. This effect was also shown by Wendler et al. [46] for cast steels of similar composition.

In the present work, EDS point analysis of the material state C70 + 8 → 30 revealed manganese fractions of 4.9 wt.% in the area of high energy input during PBF-EB (see Section 2 for further information on building conditions), whereas 6.0 wt.% was determined for the area exposed to only 30 J/mm^3^. These results indicate that an adequate adjustment of the process parameters enables chemical variations in the same part, even if it is built out of the same powder feedstock, see [19].

**Figure 5:**
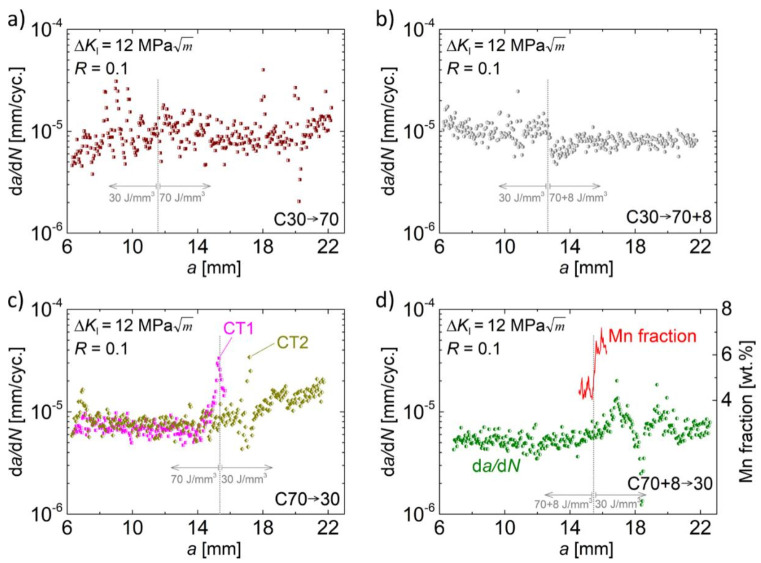
Crack growth rates d*a*/d*N* versus crack length a for the graded specimens of material states C30 → 70 (**a**), C30 → 70 + 8 (**b**), C70 → 30 (**c**), and C70 + 8 → 30 (**d**) at a cyclic stress intensity factor of 12 MPa$\sqrt{m}$. The vertical grey dotted lines indicate the gradation obtained from light microscopy of fracture surfaces. (**c**) includes two specimens marked by CT1 and CT2 and in (**d**) the manganese fraction for a limited crack length segment as determined by an EDS line scan is added.

The influence of the chemical gradation of batch C on the cyclic crack growth at a constant cyclic stress intensity factor of 12 MPa$\sqrt{m}$ is shown in Figure 5 in form of cyclic crack growth rates d*a*/d*N* plotted against the crack length a. For material state C30 → 70 (Figure 5a), the crack started in the area being exposed to low energy input during PBF-EB and propagated into the area with high energy input. Nevertheless, for the whole crack length, the crack growth rate remained constant at about 10^−5^ mm/cycle accompanied by fairly pronounced scatter. Thus, no distinct effect originating from the chemical gradation on the crack growth behavior can be deduced based on these findings. The material state C30 → 70 + 8 in Figure 5b, whose only difference to the former material state is the subsequent scanning of the already solidified material with an energy input of 8 J/mm^3^ within the high energy area, exhibited less scatter. In this case, a slight tendency towards increasing crack growth rates can be observed when the crack propagated across the interface of areas with low and high energy input (vertical dotted grey line in Figure 5b). When reaching the gradation layer, the crack growth seemed to be slightly reduced but then converged towards the initial level with further propagation. However, even if these described changes in crack growth rates were caused by the gradation of the material, the effects were relatively small and opposed (first accelerating, then decelerating), i.e., they did not markedly influence the velocity of the crack in the long term.

For crack propagation in the opposite direction with regard to the gradation, i.e., from the area of high energy to the area of low energy input (Figure 5c,d), the behavior in the range of 15 mm to 19 mm was obviously different. The curves for two CT specimens of the C70 → 30 material in Figure 5c both started at crack growth rates of about 8 × 10^−6^ mm/cycle. Then, they were characterized by a significant and steep increase at a crack length of about 15 mm (CT1) and 17 mm (CT2), respectively, followed by a similarly steep decrease. The offset in crack length (15 mm vs. 17 mm) between the two specimens is supposed to stem from the measurement method during the test with a one-sided crack gage (see Section 2). If the crack does not propagate equally on both surfaces, the measurement does not consider this effect and the effective crack length could deviate to a certain extent.

A similar behavior as described for material state C70 → 30 (Figure 5c) was observed for the material state C70 + 8 → 30 (Figure 5d). After an increase of the crack growth rate at a crack length of about 16 mm, the crack growth rate temporarily decreased down to almost 10^−6^ mm/cycle and, shortly afterwards, returned to the initial value. For the crack segment between 14.6 mm and 16.4 mm, Figure 5d additionally depicts the manganese fraction measured by an EDS line scan (red line in Figure 5d). At a crack length of 15.5 mm, a pronounced increase of the manganese fraction can be seen. This approximately corresponds to the beginning of the serrated crack growth curve. To a certain extent, potential offsets in crack length between the EDS measurement and the d*a*/d*N* curve can result from the limitations of crack length measurement during the cyclic tests for the same reason discussed above for the one-sided crack length measurement.

Taking all these findings into account, it seems likely that the accelerating and decelerating effects in the crack growth behavior of the material states C30 → 70 + 8, C70 → 30 and C70 + 8 → 30 in the direct vicinity of the interface of high and low energy area, respectively, originated from the chemical gradation of the CT specimens. However, this cannot be assessed conclusively due to the limited data. However, the available data already reveals that, even if there is an influence of gradation, it is only temporary and, furthermore, the promoted effects are opposed and more or less compensate each other. Neither a solely decelerating nor a solely accelerating influence on the crack propagation can be deduced. Thus, the gradation does not affect the crack propagation in the long run, i.e., the material behavior was more or less insensitive to chemical gradation. On the other hand, a completely different picture may arise if tests are performed at lower cyclic stress intensity factors in the near-threshold regime as the microstructure generally has a much higher impact on the crack propagation in this regime [28]. This could be the reason for the discrepancies of the present results compared to the study by Brenne et al. [26], who investigated a graded AISI 316L processed by PBF-LB. Different grain sizes and morphologies by applying different processing parameters were achieved in that study. The respective microstructural gradation significantly affected the cyclic crack growth behavior of the steel at a constant cyclic stress intensity factor of 5 MPa$\sqrt{m}$ [26]. Thus, two main differences arise in comparison to the present study: (i) a different kind of microstructural gradation and (ii) a distinctly lower stress intensity factor range. Which of these differences led to the contradictory finding of a significant influence of the gradation cannot be assessed at this time. Therefore, in future work the present investigations should be repeated at relatively low stress intensity factors.

## 4. Summary

The austenitic stainless steel X5CrMnNi16-7-6 was processed by PBF-EB and assessed in cyclic crack growth experiments. The influence of the building parameters as well as the building direction on the crack propagation rate were analyzed. Furthermore, graded specimens with high manganese fraction and low manganese fraction were manufactured to investigate the influence of a chemical gradation on the cyclic crack growth rate. The findings can be summarized as follows:

- The cyclic crack growth is not affected by the building direction of the PBF-EB process; i.e., crack propagation parallel and perpendicular to the building direction were similar due to the almost isotropic microstructure.
- The crack growth rates were not affected by different PBF-EB process parameters and, eventually, different manganese fractions.
- A compositional gradation within the same specimen did not effectively influence the crack growth rate at a cyclic stress intensity factor of 12 MPa$\sqrt{m}$. Despite some local crack growth rate changes in the direct vicinity of the interface, the crack propagation behavior was not affected in the long run.

## Figures and Tables

**Table 1:** Chemical composition and d_10_, d_50_, and d_90_ values of the steel powders. The concentration of C was determined by the combustion infrared detection technique, N by inert gas fusion infrared and thermal conductivity detection, Cr and Ni by X-ray fluorescence spectroscopy, and the other elements by inductively coupled plasma spectroscopy.

Manufacturing Technology	Cr (wt.%)	Mn (wt.%)	Ni (wt.%)	C (wt.%)	N (wt.%)	Si (wt.%)	d10 (µm)	d50 (µm)	d90 (µm)
PBF-EB	15.8	6.4	5.9	0.05	0.04	0.9	47.8	72.5	118.8
HP	16.4	7.1	6.3	0.02	0.06	0.1	12.4	25.9	46.6

**Table 2:** Overview of the homogenous and graded material states, the CGD of the associated CT- specimens in relation to the BD (vertical, bottom up), the PBF-EB parameters used and the resulting Mn content according to spark emission spectroscopy measurements. Acceleration voltage *U_A_* and layer thickness *t* were kept constant at 60 kV and 50 µm, respectively. In the upper half of the blocks of material states C30 → 70 + 8 and C70 + 8→ 30, each layer was exposed to energy input by the electron beam twice. *v_s_*: scan speed, *l*: hatch distance, *I_B_*: beam current, *E_vol_*: energy input per volume unit, exp.: exposure.

Batch	Type	CGD↔BD (↑ BD)	*E_vol_* (J/mm^3^)	*v_s_* (mm/s)	l (µm)	IB (mA)	Mn (wt.%)
A48pa	homogenous	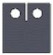	48	2500	75	7.5	4.4
A48pe		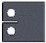	48	2500	75	7.5	
A30pa		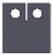	30	4000	75	7.5	4.0
A30pe		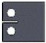	30	4000	75	7.5	
B50pa		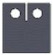	50	2000	90	7.5	4.5
B50pe		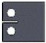	50	2000	90	7.5	
B33pa		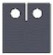	33.3	3000	90	7.5	5.2
B33pe		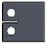	33.3	3000	90	7.5	
C30 → 70	graded	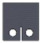	70.6	1700	75	7.5	2.9
			30	4000	75	7.5	5.1
C70 → 30		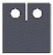	70.6	1700	75	7.5	4.0
			30	4000	75	7.5	5.2
C30 → 70 + 8		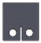	1st exp.: 70.6 2nd exp.: 8	1700 5000	75 15	7.5 5	3.1
			30	4000	75	7.5	5.2
C70 + 8 → 30		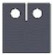	1st exp.: 70.6 2nd exp.: 8	1700 5000	75 15	7.5 5	3.8
			30	4000	75	7.5	5.2

## Data Availability

The data presented in this study are available on request from the corresponding author.
